# Mastcam Image Resolution Enhancement with Application to Disparity Map Generation for Stereo Images with Different Resolutions

**DOI:** 10.3390/s19163526

**Published:** 2019-08-12

**Authors:** Bulent Ayhan, Chiman Kwan

**Affiliations:** Applied Research, LLC, Rockville, MD 20850, USA

**Keywords:** image resolution, image enhancement, Mastcam, disparity map, depth map

## Abstract

In this paper, we introduce an in-depth application of high-resolution disparity map estimation using stereo images from Mars Curiosity rover’s Mastcams, which have two imagers with different resolutions. The left Mastcam has three times lower resolution as that of the right. The left Mastcam image’s resolution is first enhanced with three methods: Bicubic interpolation, pansharpening-based method, and a deep learning super resolution method. The enhanced left camera image and the right camera image are then used to estimate the disparity map. The impact of the left camera image enhancement is examined. The comparative performance analyses showed that the left camera enhancement results in getting more accurate disparity maps in comparison to using the original left Mastcam images for disparity map estimation. The deep learning-based method provided the best performance among the three for both image enhancement and disparity map estimation accuracy. A high-resolution disparity map, which is the result of the left camera image enhancement, is anticipated to improve the conducted science products in the Mastcam imagery such as 3D scene reconstructions, depth maps, and anaglyph images.

## 1. Introduction

Due to hardware limitation in data storage, scarce bandwidth in data downlink and cost of sensor, different imagers have chosen to have different priorities with respect to image resolution. As an example, onboard the Mars rover Curiosity, there are two mast cameras (Mastcam), which act as eyes for the rover [1]. The Mastcams have different spatial resolutions and is a perfect example for demonstrating the fusion of two images with different resolutions. The left Mastcam has three times wider field of view than that of the right. However, the right Mastcam has three times better resolution. Although the two cameras work independently, stereo images can still be formed from them for the Mastcam image pairs that have overlapping views [2]. 

There are several research papers that discuss about using Mastcams for anomaly detection, rock composition estimation, classification and finding interesting destinations for the rover. In [3], the right and left Mastcam band images which have different wavelengths have been registered, and the stacked and co-registered bands are used for anomaly detection. In [4], both the left and right Mastcams images are used to generate a set of multispectral signatures for a few selected pixel locations, and these multispectral signatures are used to investigate the composition and mineralogy of materials. There is also a growing interest in adapting augmented and virtual reality tools to Mars rover missions [5,6,7]. As an example, NASA and Microsoft have developed a software called OnSight that enable scientists to work virtually on Mars using wearable technology called the Microsoft HoloLens [8]. The OnSight software uses imagery acquired by the Curiosity rover and creates a 3D model of the Mars terrain. This enables the users and scientists to explore the actual dunes and valleys explored by the rover in 3D creating the feeling for scientists as if they are in the field. The 3D scene reconstruction using rover imagery including Mastcam stereo images can thus have both an educational and scientific impact for current and future Mars exploratory missions. The disparity maps are important since they provide depth information from the 2D stereo images. In recent years, disparity map estimation using monocular images was also studied in a number of works [9,10,11]. In these works, the objective is to estimate the disparity map from a single-color image only using deep learning architectures. This is a more challenging research problem in comparison to estimating disparity maps from stereo image pairs since there is a single image instead of an image pair to estimate the disparity map. Disparity maps are utilized in 3D reconstruction, robot navigation, obstacle avoidance, and target tracking. Due to the differences in image resolutions of the Mastcam images, a generic disparity map estimation using the original Mastcam images may not result in full utilization of the high-resolution image that is available only in the right Mastcam but is dependent on the low-resolution left camera image. A more accurate and detailed 3D scene reconstruction could be possible if a high-resolution disparity map is available. 

In this paper, we introduce an in-depth application of image resolution enhancement to the low-resolution left camera image in the Mastcam image pair and evaluate the impact of the left camera image enhancement on the disparity map estimation quantitatively. The motivation of this work is to have a disparity map in higher resolution than before by enhancing the low-resolution image in the image pair and estimating the disparity map using this enhanced left image and the high-resolution right image. To the best of our knowledge, even though stereo images had been generated using the Mastcam instruments [12], examining the impact of the image enhancement on the disparity map estimations has not been studied and we consider this paper as a first along this direction.

For the low-resolution left camera image enhancement, we used the bicubic interpolation [13] as the benchmark technique. A second investigated image enhancement method [2,12] is an adaptation of the two-step image registration technique in [3] with pansharpening [14,15,16,17,18]. We will call this method the pansharpening-based image enhancement method in this paper. Unlike bicubic interpolation, the pansharpening-based image enhancement method requires the stereo image pair for enhancement. In recent years, a significant amount of research work had been published using deep learning-based image super resolution techniques [19,20,21]. For the third investigated enhancement method, we used a deep-learning based method, which is known as enhanced deep super resolution (EDSR) [22]. After the left camera image enhancements with these three methods, the disparity maps are estimated using the enhanced left camera images and the impact of the left camera image enhancement on the disparity map estimations are examined quantitatively.

This work demonstrates that a high-resolution disparity map estimation is possible for stereo image pairs with different camera resolutions such as the case in Mars Curiosity rover’s Mastcams. This is the main contribution of this work. It is shown that this can be achieved by enhancing the low-resolution left camera image. The right camera image content is utilized for enhancement with the pansharpening-based method and EDSR. For the demonstrations, 20 Mastcam stereo image pairs and a stereo image pair from the Middlebury dataset were used. Among the three investigated methods, overall, the deep learning-based method, EDSR, is found to perform better than others in the image enhancement and in accurate disparity map estimation performance. The extensive comparison study using actual Mastcam imagery in this paper is considered as another contribution of this work. 

In Section 2, a brief information is provided about the Mastcam instruments of the Curiosity rover and the different resolutions of the left and right Mastcams. In Section 3, we introduce the three image enhancement methods. In Section 4, the disparity map estimation processing steps applied in this work are outlined. Section 5 summarizes the low-resolution image enhancement and disparity map estimation results and observations for a Middlebury stereo image pair [23]. Section 6 corresponds to the results and analyses when 20 Mastcam image pairs from the Mars Curiosity rover were used for the left camera image enhancement and disparity estimation using the enhanced left camera images. A total of six different image quality measures and an average absolute error measure for disparity map assessment are used in the performance comparisons of the three methods. Some practical issues such as the impact of image registration on the disparity map estimates of the pansharpening-based method are discussed in this section as well. Finally, some concluding remarks are highlighted in Section 7.

## 2. Mastcam Imagery for High Resolution Stereo Image and Disparity Map Generation

**Figure 1:**
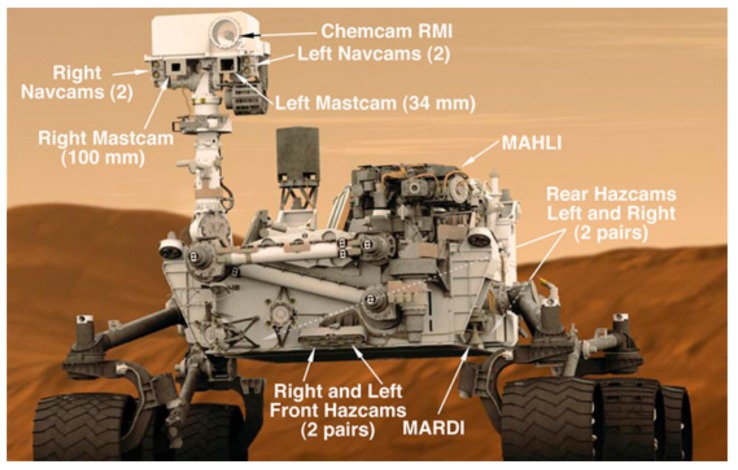
Mars rover Curiosity and its onboard cameras [24]. Mastcam imagers act as eyes of the rover for rock sample selection and rover guidance.

The two Mastcams of Curiosity are shown in Figure 1. There are several other imagers for navigation, landing (MARDI), obstacle avoidance (Hazcams and Navcams), chemical composition analysis (Chemcam). The left Mastcam imager has three times lower resolution than that of the right. The left is usually used for long range image acquisition and the right camera is for near field data collection.

The Mastcams are multispectral imagers with nine bands in each. Here, we only focus on the use of the RGB bands for stereo image and disparity map generations. The left and right cameras of the Mastcam imager have different resolutions. Moreover, the cameras are not calibrated for stereo image formation, as they normally work independent of each other. To generate stereo images from these two cameras with different resolutions, a common practice is to downsample the high-resolution right camera image to the same as the lower one. After that, the stereo images are formed by following some standard procedures. It is well known that the downsampling of the high-resolution camera image is more economical but less effective, as the resulting stereo images will have lower resolution. The resulting disparity map derived from the resulting stereo images also has a low resolution. The motivation of this work is to improve the disparity map estimation by enhancing the low-resolution image and then estimating the disparity map using the enhanced left camera image and the right camera image.

## 3. Image Enhancement Methods 

We briefly introduce the three image enhancement methods applied to enhance the low-resolution left camera image in this work. These three methods are:

- Bicubic interpolation [13];
- Pansharpening-based method [2,12];
- EDSR (Deep-learning based image super resolution method) [22].

### 3.1. Bicubic Interpolation 

Bicubic interpolation is provided as an image resizing tool in image editing programs like Photoshop [25]. In image resizing with bicubic interpolation, the color information of the to-be inserted pixels is approximated using the values of the surrounding pixels, which is the 4x4 neighborhood of the pixel. In the approximation, closer pixels are given a higher weighting. Even though bicubic interpolation preserves fine detail and sharpness better than the bilinear interpolation algorithm, it might create image artifacts like blurring or zigzag on edges [26]. Bicubic interpolation is used as the benchmark method in this paper. One can refer to [13] for other technical details of bicubic interpolation.

### 3.2. Pansharpening-Based Image Enhancement Method

**Figure 2:**
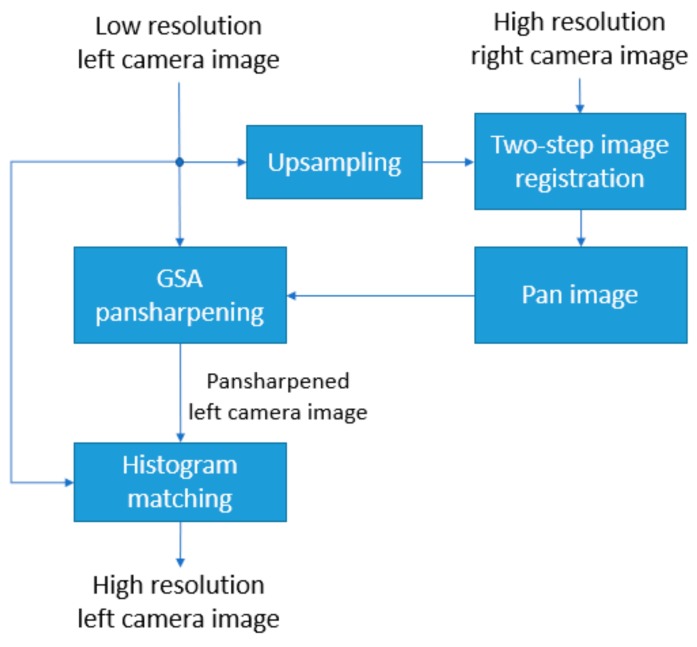
Block diagram of the pansharpening-based image enhancement method.

**Figure 3:**
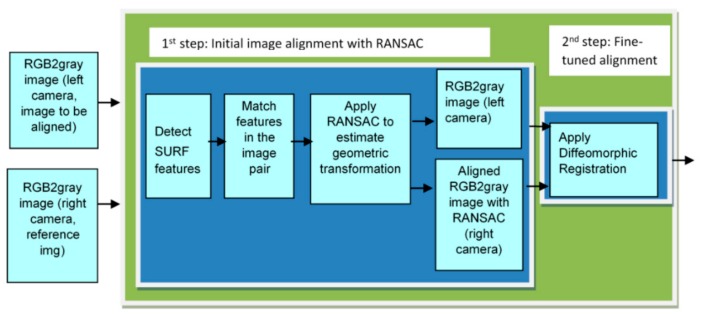
Block diagram of the two-step image registration technique [3].

The pansharpening-based image enhancement method [2,12], of which its block diagram can be seen in Figure 2, is the adaptation of the two-step image registration technique [3] with pansharpening. Pansharpening is the common name given to the process of merging high-resolution panchromatic and lower resolution color images to create a high-resolution color image. In the pansharpening-based image enhancement method, the right camera image is aligned to the upsampled left camera image using the two-step image registration technique [3]. The block diagram of the two-step image registration technique can be seen in Figure 3. The first step of the two-step image registration technique is a coarse alignment of the right camera image to the upsampled left camera image using the RANSAC (Random Sample Consensus) technique [27] with speeded up robust features (SURF) [28] extracted from the stereo image pair. SURF features are scale and rotation invariant interest points and extensively used to find correspondences in image pairs with the same scene [28]. The second step of the two-step image registration technique [3], which is known as diffeomorphic registration, fine-tunes the first step’s coarse registration. 

The aligned right camera image from the diffeomorphic registration step of the two-step image registration technique becomes the high-resolution panchromatic (pan) image and it is used to enhance the low-resolution left camera image using the Gram–Schmidt Adaptive (GSA) pansharpening technique [29]. Different from [2,12], in this work, a histogram matching processing step [30] is incorporated to the pansharpening-based image enhancement method to match the histogram of the pansharpened left camera image to the histogram of the low-resolution left camera image as can be seen in the block diagram in Figure 2. It is observed that this step improves the scores of some of the image quality measures such as peak signal to noise ratio (PSNR) and root mean Square error (RMSE). 

### 3.3. EDSR (Deep Learning-Based Super Resolution Method)

**Figure 4:**
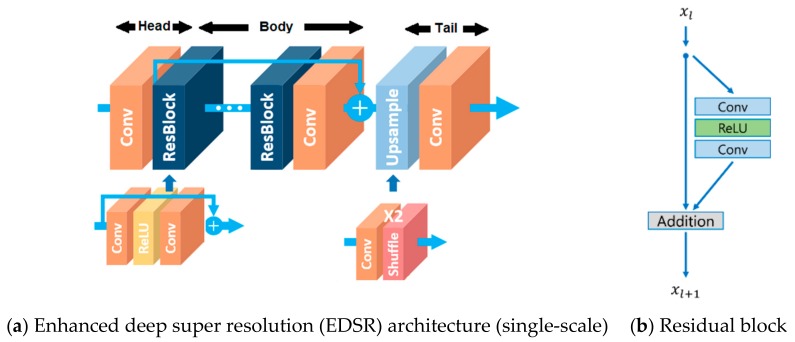
Single scale EDSR architecture and a residual block in EDSR [22].

In recent years, image super resolution using deep neural networks, also known as deep learning, has gained a lot of attention [19,20,21,22]. Through extensive training with many images, deep learning can achieve good performance. One advantage of deep learning methods is that, once the model is learned, it can be used to enhance a given low resolution image without a panchromatic band. EDSR is one of these deep-learning methods which gained reputation by winning the 2017 super resolution challenge [22]. The deep learning network architecture in EDSR has similarities with the conventional ResNet architecture. However, some common ResNet architecture modules, which are deemed as unnecessary by the authors, are removed from the EDSR architecture [22]. The EDSR architecture (single scale baseline) can be seen in Figure 4a. The body of the EDSR architecture consists of several residual blocks (ResBlock). A single residual block architecture can be seen in Figure 4b. 

The single-scale baseline EDSR architecture is used in this work. The components of the EDSR baseline architecture and the parameters for these components can be seen in detail in Table 1. A total of 16 residual blocks and 64 feature maps are used in the baseline EDSR architecture.

**Figure 5:**
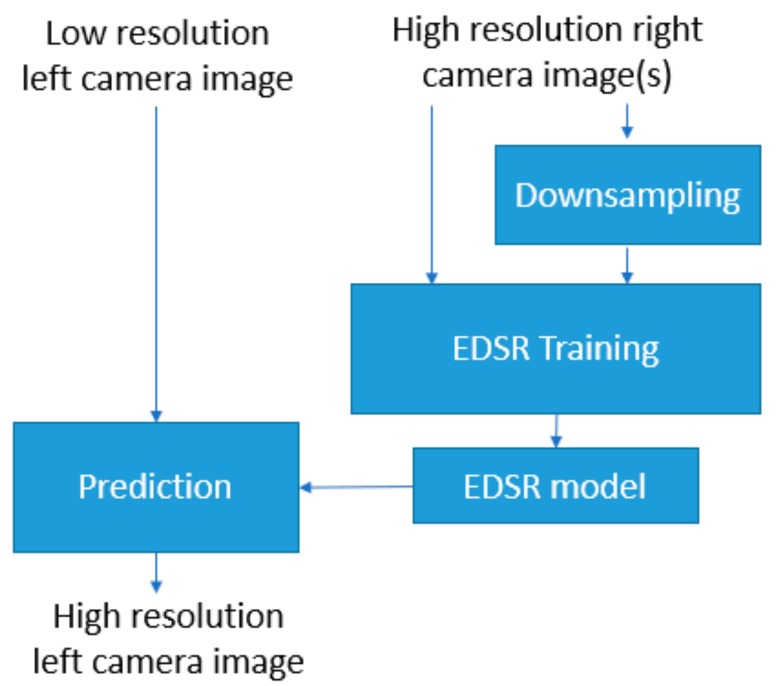
Processing steps of EDSR (training and testing).

When training an EDSR model for Mastcam images, the corresponding high-resolution right camera images of the test images (low-resolution left camera images) are used in the training dataset. The down-sampled high-resolution right camera images at the image resolution scale of interest, which is two (×2), are used in training a model as well. Once the EDSR model is trained, the testing is straightforward by feeding the test low-resolution left camera image into the deep learning network with the trained EDSR model. The simplified block diagram of the processing steps of EDSR is shown in Figure 5.

## 4. Disparity Map Estimation

**Figure 6:**
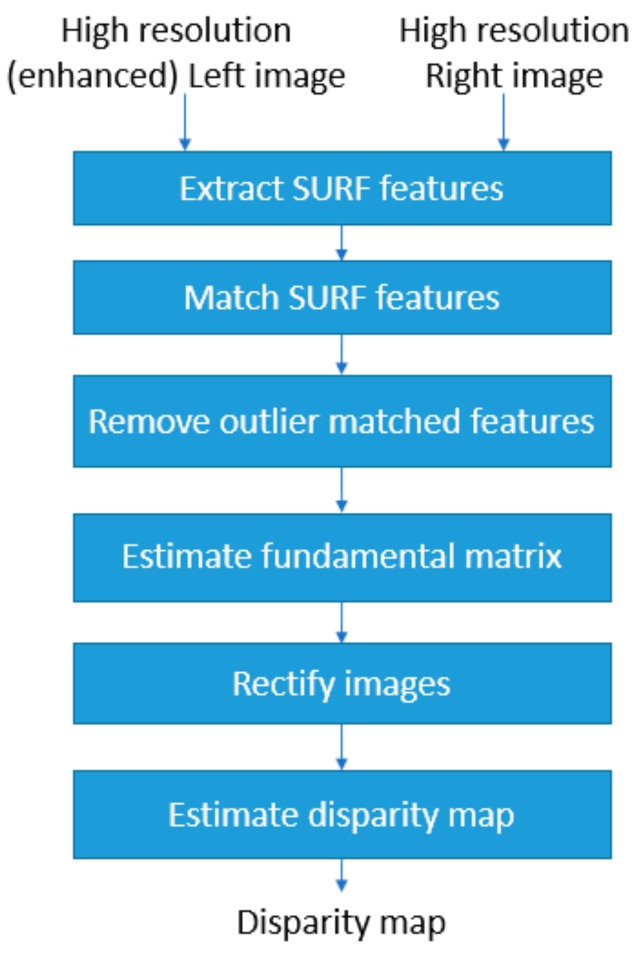
Processing steps of the disparity map estimation.

In this section, we outline the processing steps to estimate the disparity map using the enhanced left camera image and the right camera image. A block diagram of the disparity map estimation processing steps can be seen in Figure 6. A disparity map, which is used for depth detection, consists of the distance information between the matched points in the stereo image pair. Overall, the disparity map can be thought of an intensity image with brighter pixels in the map denoting lesser depth and greater distance (or motion) between the matched points with respect to the brighter pixel area in the stereo image pair. Darker pixels in the disparity map correspond to greater depth and smaller distance (or motion) between the matched points with respect to the darker pixels area. In the disparity map estimation, for each point in the left image, the corresponding point in the right image is found. This is a challenging task and known as the correspondence problem [31]. In this work, to estimate the disparity map, an image rectification process is applied on the enhanced left camera image and the right camera image. Matching between the points can be done more efficiently in accurately rectified image pair since the matching becomes a one-dimensional search after rectification [31]. The image rectification process consists of a number of processing steps, one of which is the fundamental matrix estimation. The fundamental matrix is necessary for the rectification transformations. For fundamental matrix estimation, SURF features [28] are extracted from the stereo image pair and the extracted SURF features from each image are matched. The SURF feature pairs that do not meet the epipolar constraint are removed from the fundamental matrix estimation. In a stereo image pair, for each point in one of the stereo images, there is a corresponding epipolar line in the other image. Suppose ***x*** is a point in the stereo image of the first camera, for this point, the epipolar line in the second stereo image can be considered as the projection of the ray from point ***x*** to the center of the first camera to the second camera image. Any point ***x’*** in the second stereo image that matches to the first camera image must be on this epipolar line [32] and this is the epipolar constraint. The mapping from points in one stereo image to the epipolar lines in the second stereo image is represented by the fundamental matrix [32]. After transformation with the fundamental matrix, the resultant rectified left and right camera images are used to the estimate the disparity map. In the disparity map estimation, the semi-global block matching method [33] is applied to the rectified image pair. This method computes disparity by comparing the sum of absolute differences (SAD) of each block of pixels in the image while also checking similar disparity on the neighboring blocks [33]. 

## 5. Results and Analyses for a Middlebury Stereo Image Pair

The low-resolution camera image enhancement and disparity map estimation are first demonstrated using an image section from the motorcycle image pair in the Middlebury stereo dataset [23]. The technical specifications of the cameras used for capturing of the motorcycle image pair can be found in [23]. This image pair is used to show that a high-resolution disparity map can be estimated for a hypothetical case in which one of the stereo camera images has lower resolution than the other camera image in the pair.

### 5.1. Low-Resolution Left Camera Image Enhancements 

To imitate the different resolution stereo image pair scenario in the Middlebury motorcycle stereo image pair, the original left camera image is intentionally down-sampled four times (×4) and this down-sampled image is used for image enhancement with the three methods. It is worth mentioning that since the left and right camera images in the Middlebury stereo image pair are already rectified, no rectification process is applied and the disparity map is estimated directly from the stereo image pair. For the EDSR model training, no custom EDSR model is trained and the authors’ baseline training model for a scale of four (×4) [22] is used.

**Figure 7:**
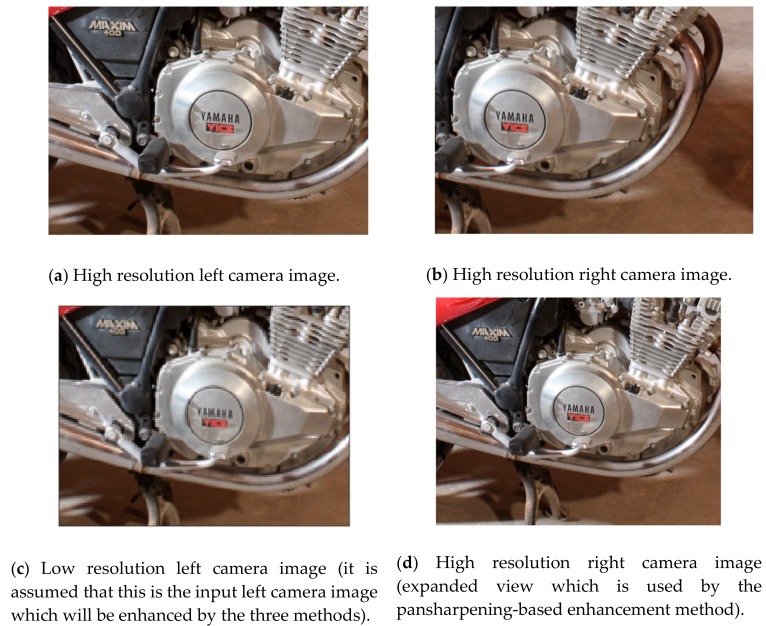

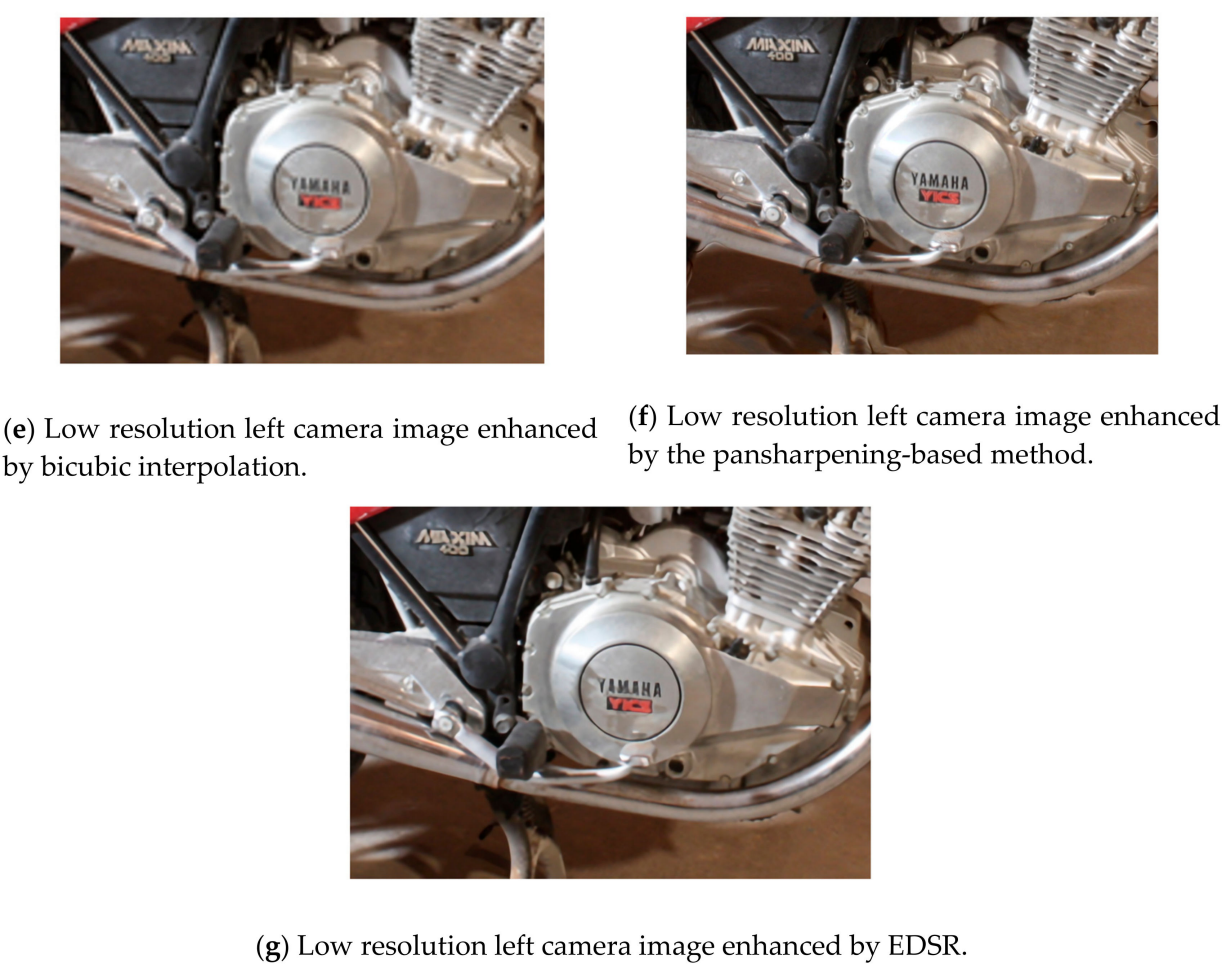
A section of the motorcycle stereo image pair and the enhanced left camera images with the three methods.

The high resolution left and right camera images of the motorcycle image pair are shown in Figure 7a,b, respectively. Figure 7c shows the hypothetical low-resolution left camera image (which is the four times down-sampled version of the original high-resolution left camera image). An expanded right camera image is shown in Figure 7d which is used by the pansharpening-based image enhancement method when aligning the right camera image to the left camera image. Figure 7e–g show the resultant left camera image enhancements with the three methods. According to the results in Figure 7, the enhanced left camera image by EDSR is found to be visually quite appealing. For assessing the image quality of the enhanced left camera images with the three methods, we applied five image quality measures. These are root mean square error (RMSE), structural similarity index measure (SSIM) [34], peak signal to noise ratio (PSNR), human visual system (HVS) [35] and human visual system with contrast sensitivity function and noise masking (HVSm) [36]. 

When computing the image quality measures, we used the original high-resolution left camera image as the reference (ground truth) image. The grayscale versions of the images are used when computing SSIM and RMSE scores. Table 2 summarizes the resultant five measures for the three methods. It is worth mentioning that higher values in SSIM, PSNR, HVS, and HVSm, and lower values in RMSE correspond to better image quality. The results in Table 2 clearly show that EDSR provides the best enhancement performance in all five measures followed by the pansharpening-based method. Bicubic interpolation performs the worst among the three. 

### 5.2. Disparity Map Estimation

**Figure 8:**
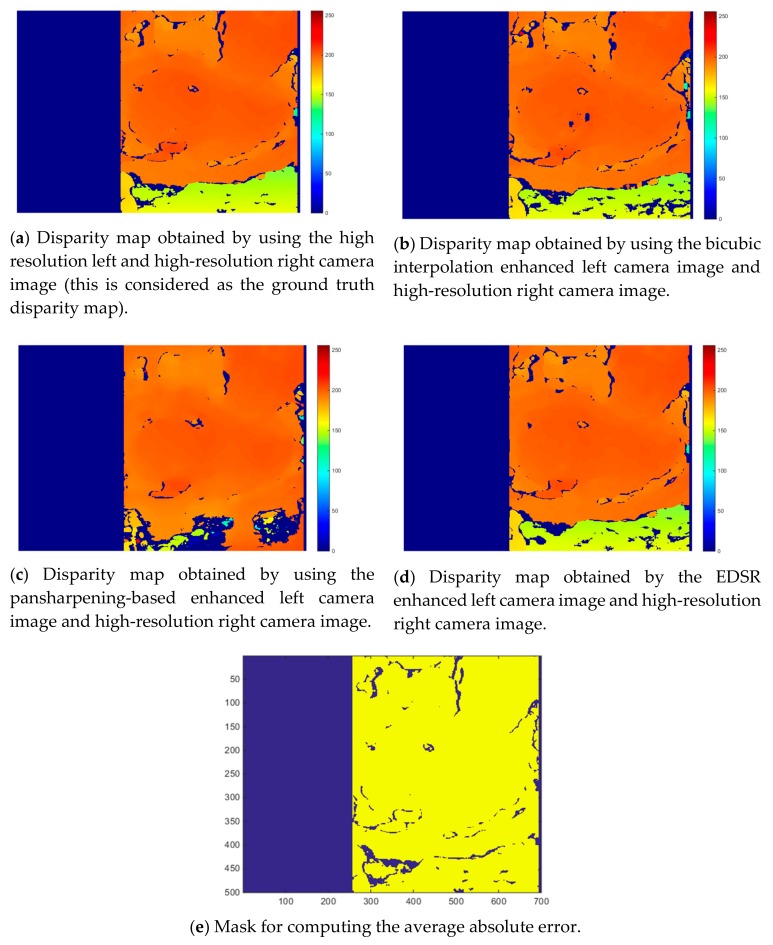
Disparity map estimations with the three methods and the mask for computing average absolute error.

After generating the enhanced left camera images with the three methods, the disparity maps are estimated using the enhanced left camera images and the original right camera image. Figure 8a corresponds to the disparity map obtained by using the original left camera image. We use this disparity map as the ground truth disparity map when conducting performance assessments. 

For comparing the disparity map estimation performances, an “average absolute error” measure is used to compare the accuracy of the estimated disparity maps of the three methods with respect to the ground truth disparity map. The ground truth disparity map is obtained using the original left and right camera images. Suppose $\left\{g_{1},g_{2},\ldots ,g_{N}\right\}$ is the set of ground truth disparity map pixel values that are used for performance assessment of the three methods where *N* is the total number of pixels in *D*. The location of the pixels in $\left\{g_{1},g_{2},\ldots ,g_{N}\right\}$ correspond to the overlapping area of the compared disparity maps from the three different image enhancement methods. Suppose $\left\{b_{1},b_{2},\ldots ,b_{N}\right\}$ is the set of the corresponding *N* disparity map pixel estimates for the applied method *B*. The "average absolute error” for method *B* is computed as: $\left(\sum_{i=1}^{N}abs(g_{i}-b_{i})\right)/N$.

Figure 8b–d show the resultant disparity maps for the three methods. Additionally, in Figure 8e the mask used for computing the average absolute error in the disparity maps is shown. Only the pixels highlighted in this mask are used in the average absolute error computation. Since the left and right camera images have different views, the overlapping scene from the left and right camera images, thus the disparity map, is smaller than the original size of the enhanced left camera image. When the disparity maps from the three methods are visually examined, it can be noticed that the bottom part of the pansharpening-based method’s estimation quite differs from the ground truth disparity map. 

Table 3 shows the resultant average absolute error values for the disparity map estimations with the three methods. The average absolute error is found to be the lowest for EDSR. It is noticed that the average absolute error for the pansharpening-based method is significantly high (meaning it is worse) when compared to the other two methods. This was also visually noticed from the disparity map estimation plot in Figure 8c.

**Figure 9:**
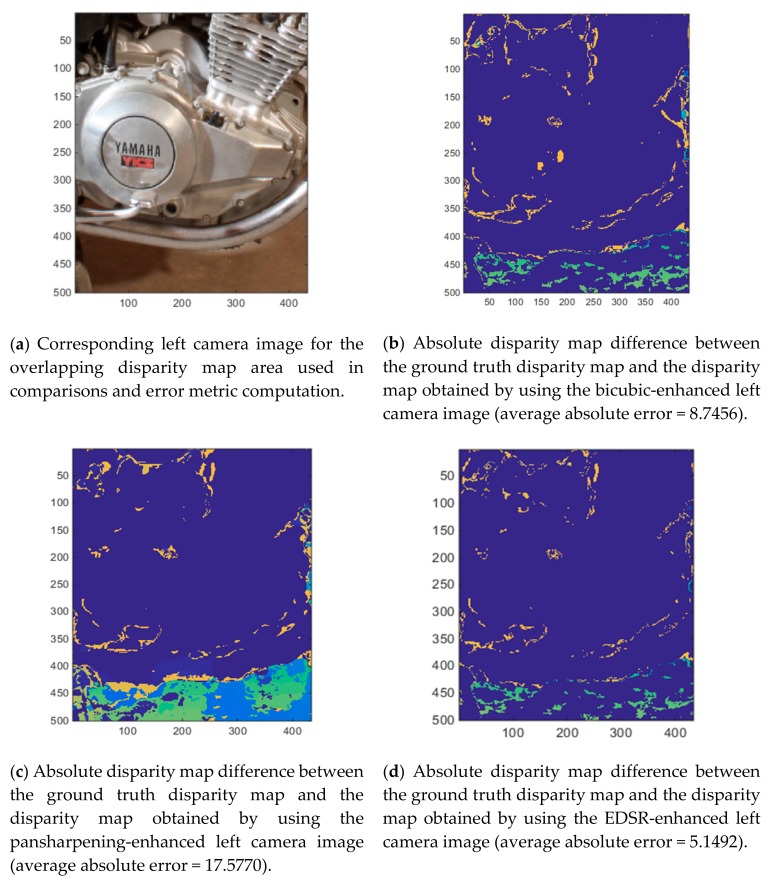
Absolute disparity map differences between the ground truth disparity map and the disparity maps obtained with the three methods and the corresponding left camera image for the overlapping disparity map area.

**Figure 10:**
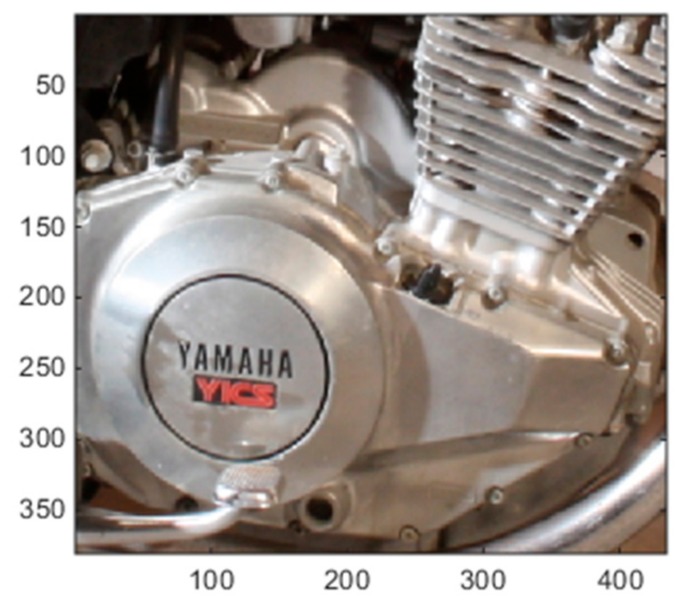
Foreground image section of the motorcycle image.

To better visualize the differences in the estimated disparity maps of the three methods with respect to the ground truth disparity map and see how the errors relate to the scene, we generated four plots as shown in Figure 9. Figure 9a shows the left camera image for the considered overlapping disparity map pixels. Figure 9b–d show the absolute disparity map difference between the ground truth disparity map and the disparity map obtained with using any of the three investigated methods. When the ground truth disparity map and the disparity map estimated by the pansharpening-based method are compared (Figure 9b vs Figure 9c), some significant differences can be observed in the bottom part. When the motorcycle image section that corresponds to the disparity map area, which can be seen in Figure 9a, is examined, it can be seen that the bottom part corresponds to the background section of the image. Due to camera view differences in the left and right images for the background area, the pixel registration performance of the two-step image registration technique used within the pansharpening-based method is poor for the background section and is relatively better in the foreground section of the image. We repeated the image quality score and average absolute error for disparity map estimation computations for the foreground part of the image only. This image part can be seen in Figure 10. Table 4 shows the five image quality measure values and Table 5 shows the average absolute error values for the disparity map estimation with the three methods for the foreground image section. As can be seen in Table 5, the average absolute error for the disparity map estimation with the pansharpening-based method is quite low for the foreground image section. Even though the pansharpening-based method still cannot outperform EDSR, it performs better than the bicubic interpolation both in the left camera image enhancement scores and also in the disparity map estimation when the foreground image is used only. 

## 6. Results and Analyses for Mastcam Stereo Image Pairs

### 6.1. Original Left Mastcam Image Enhancements

We enhanced the 20 original low resolution (LR) left camera images with the three methods. The list of the Mastcam image pairs used in this study can be seen in Table 6. These Mastcam image pairs can be downloaded from the website in [37] and the Mastcam specifications used for capturing these images can be found in [38]. 

**Figure 11:**
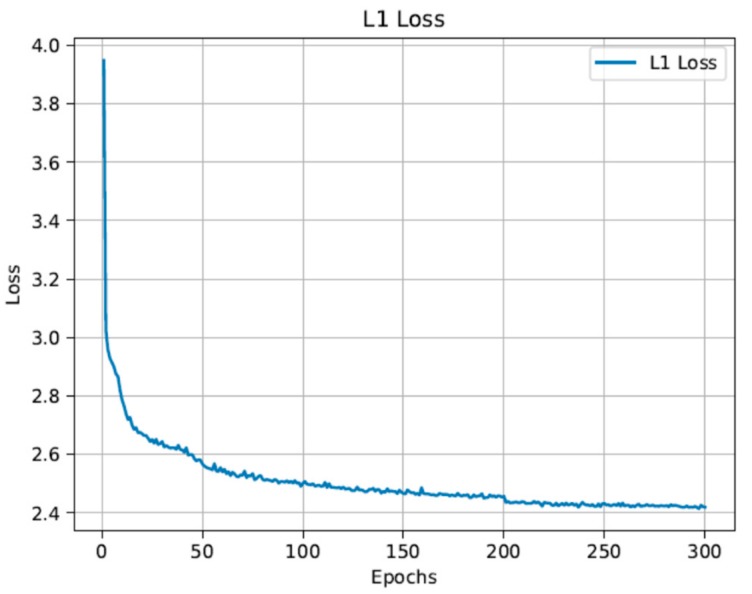
The loss plot of the training for 300 epochs.

For EDSR, a custom EDSR baseline model (×2 scale) with 16 residual blocks was trained for 300 epochs. The 20 training images included in the training data set correspond to the high-resolution right camera images of the 20 low-resolution left camera images that are going to be enhanced. The loss plot from the EDSR training can be seen in Figure 11. 

**Figure 12:**
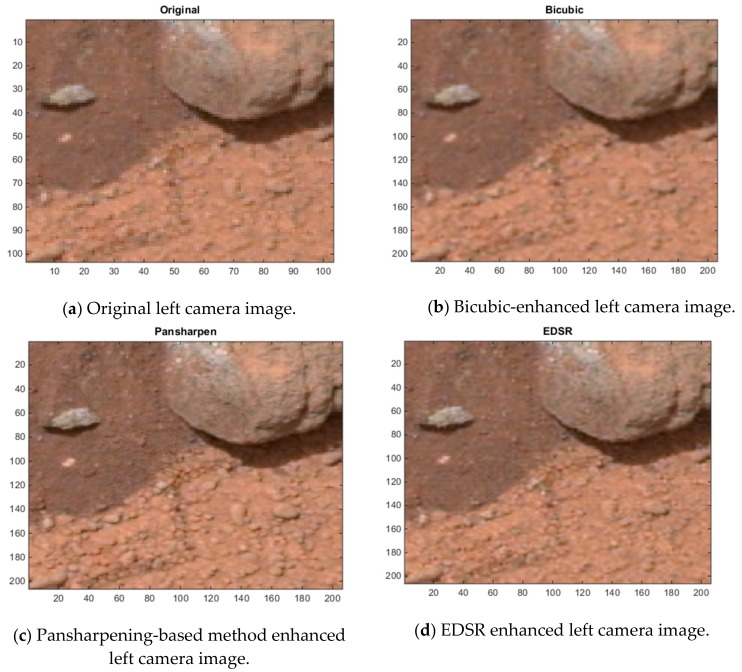
Image enhancements on 0174ML0009350000105177E01_DRCX_0PCT.png.

**Figure 13:**
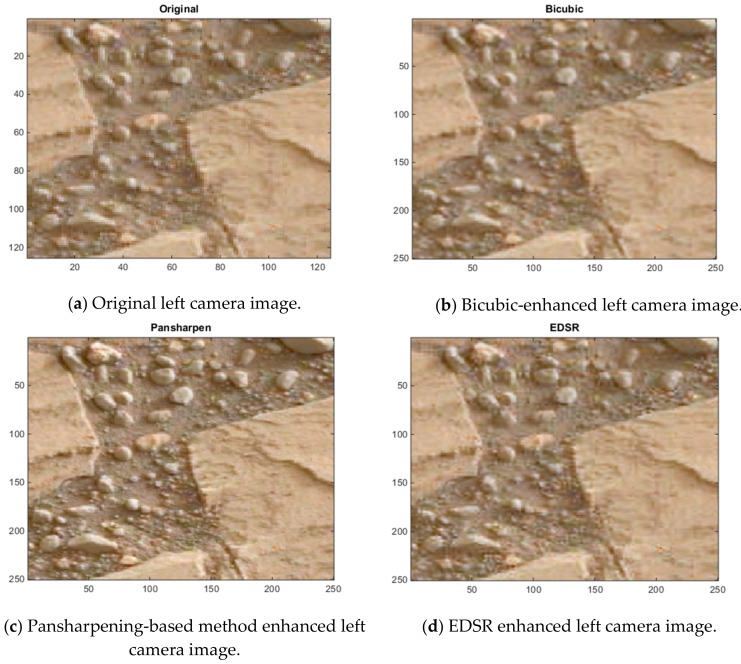
Image enhancements on 0803ML0035130050400877E01_DRCX_0PCT.png.

**Figure 14:**
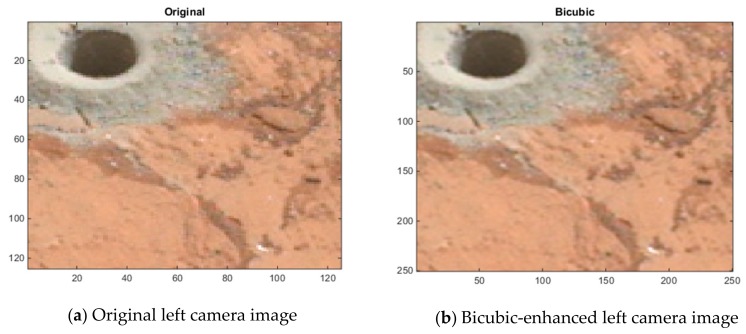

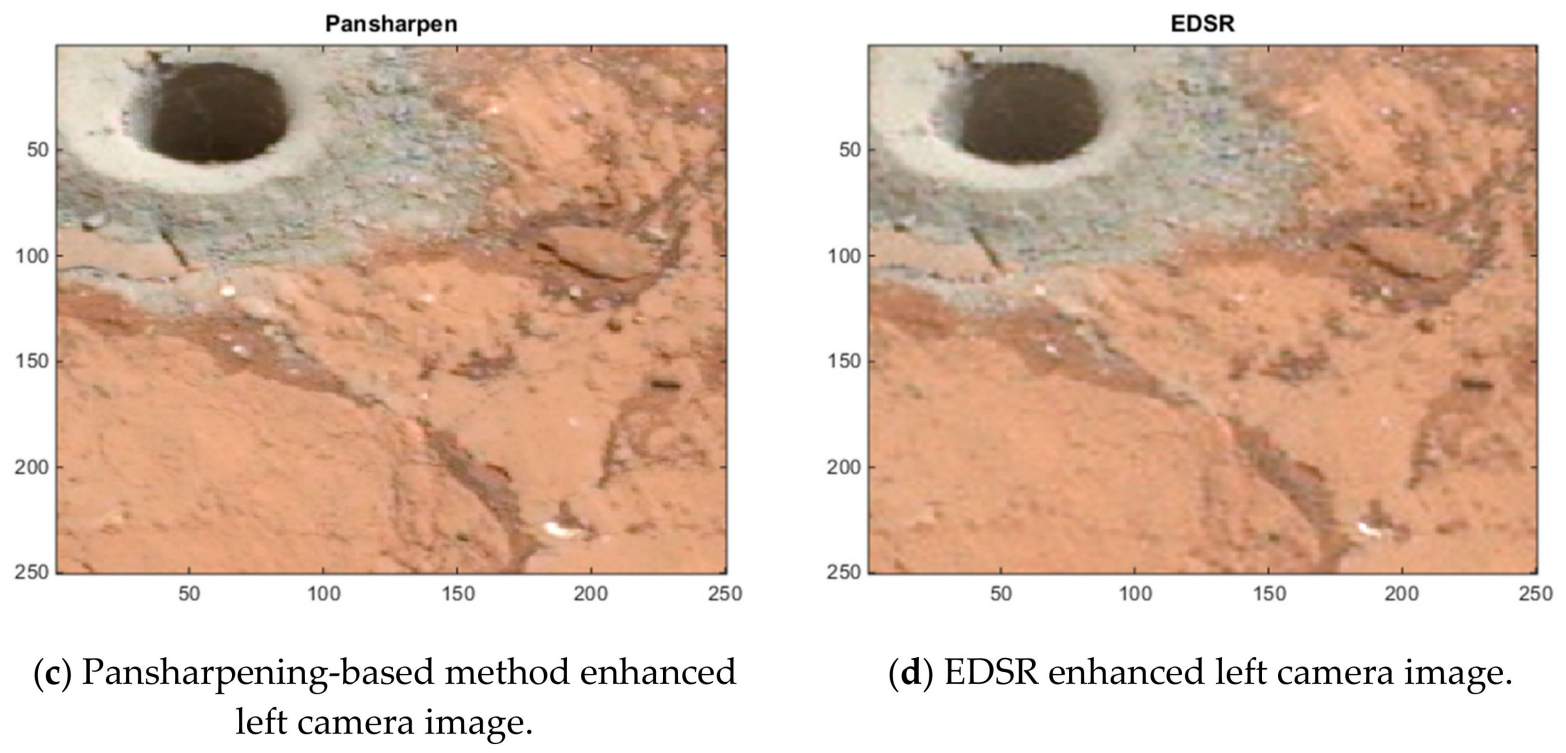
Image enhancements on 0183ML0009930000105284E01_DRCX_0PCT.png.

Figure 12, Figure 13 and Figure 14 show some cropped sections of three of the 20 left camera images (low resolution) and the resultant bicubic-enhanced, pansharpening-enhanced and EDSR-enhanced left camera images for the same image sections. Considerable improvements in the image quality can be noticed with the EDSR and especially with the pansharpening-based method when examined closely. 

**Figure 15:**
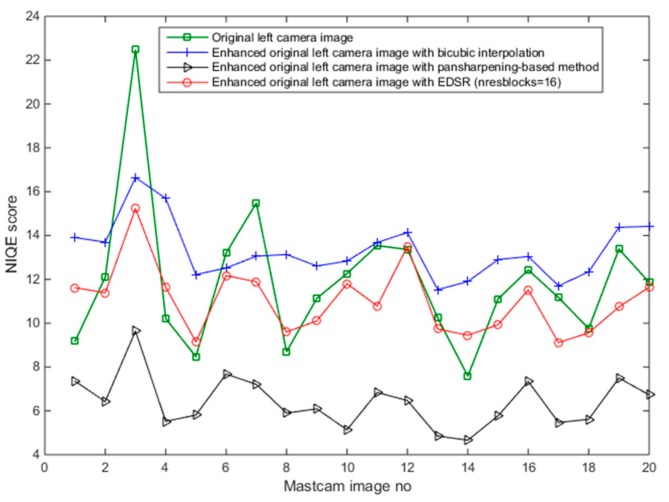
Natural image quality evaluator (NIQE) metric results for enhanced “original left Mastcam images” (scale: ×2) by the bicubic interpolation, pansharpening-based method, and EDSR (nresblocks = 16).

**Figure 16:**
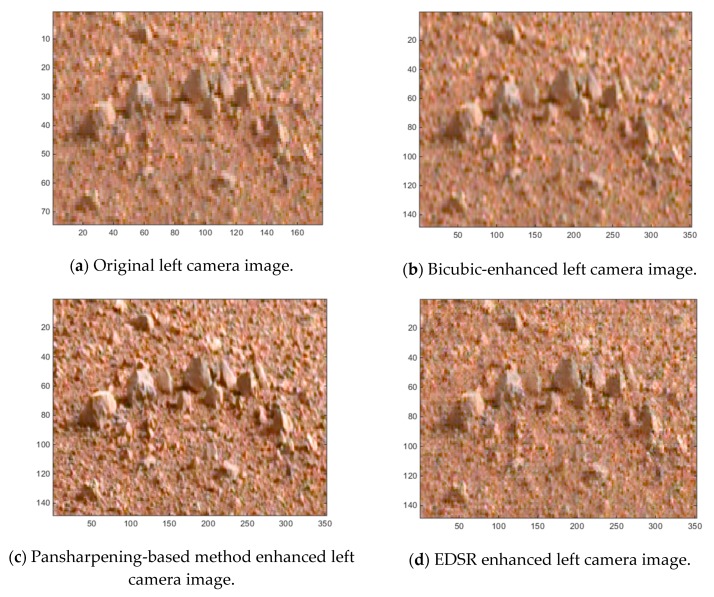
Image enhancements on the third image (0023ML0001140700100703C00_DRCX_0PCT.png).

Since the original left camera images in the 20 Mastcam image pairs are enhanced, there are no ground truth images to assess these enhanced images by the three methods. For this reason, a blind image quality assessment, natural image quality evaluator (NIQE) [39] is used instead. It should be noted that the lower the NIQE metric, the better the image quality is. Figure 15 shows the resultant NIQE values of the three investigated methods for the 20 enhanced original Mastcam left camera images. Additionally, in Figure 15, the NIQE scores of the original left camera images are included as well as a basis. According to the NIQE results in Figure 15, the pansharpening-based method outperforms the two methods and EDSR performs better than the bicubic interpolation. Moreover, the enhanced original left camera images by the pansharpening-based method yield significantly better NIQE scores than the original left camera images. This can also be visually noticed from the cropped image sections in the three image pairs in Figure 12, Figure 13 and Figure 14. The pansharpening-based method generates visually more appealing images than the other methods. The third image in the plot in Figure 15 is quite interesting in the sense that the NIQE score difference between the original left camera image and the enhanced original left camera image by the pansharpening-based method is the largest. Figure 16 corresponds to a small section of this third image and its enhanced versions by the three methods. It can be seen that some small rocks which cannot be even noticed in the original left camera image can be easily seen in the enhanced left camera image by the pansharpening-based method. The pansharpening-based method in a way brings new information to the enhanced image since it exploits the aligned high-resolution right camera image in its enhancement. This explains why the NIQE scores are extremely good with the pansharpening-based method. 

It is however worth mentioning that even though the pansharpening-based method provides the lowest NIQE values (best performance) and provides visually very appealing enhanced images, it is noticed that some pixel regions in the enhanced images do not seem to be registered in the sub-pixel level. Since the NIQE metric does not take into consideration issues related to registration in its assessment, it clearly favors the pansharpening-based method over others. 

### 6.2. Disparity Map Estimation Using Enhanced Downsampled Left Mastcam Images 

Similar to the investigation with the motorcycle image pair, the left camera images in the 20 Mastcam image pairs are intentionally down-sampled by two times (×2) and the down-sampled left camera images are enhanced with the three methods and the disparity maps are estimated using the enhanced left camera images and right camera images. This enabled assessing the image enhancement performances with image quality measures such as PSNR, RMSE since the original left camera images are used as the ground truth image. Moreover, the disparity map estimation performances are also evaluated since the disparity map which is estimated using the original left camera image is considered as the ground truth disparity map. 

Regarding the EDSR method in this investigation, we fine-tuned its architecture with respect to the number of residual blocks to see which EDSR architecture would perform better. We considered the number of residual blocks for fine-tuning since the residual blocks make the most crucial parts in EDSR’s architecture. We trained three other EDSR models with four, eight and 32 residual blocks for scale two (×2). We used the same 20 high resolution right Mastcam images in training these EDSR models for 300 epochs. With the previously trained EDSR model of 16 residual blocks, there is a total of four different EDSR models. We enhanced the “down-sampled (×2) left camera images” with these four EDSR models. We then applied five image quality measures to the 20 enhanced images from four EDSR models. Table 7 shows the average of the image quality measures for the 20 enhanced “down-sampled (×2) left” camera images with the four EDSR models. It can be seen from Table 7 that among the four EDSR models, the one with eight residual blocks performs better than the other three EDSR models. For this reason, we used the model with eight residual blocks when using EDSR for enhancing the down-sampled left camera images.

In the disparity map estimation investigation, we first used the 20 original left camera images to generate ground truth disparity maps that will be used in performance comparisons with the average absolute error measure. We then intentionally down-sampled the original left camera images by a scale of two (×2) and applied the bicubic interpolation, pansharpening-based method, and EDSR (nresblock = 8) to enhance these downsampled left camera images. We then estimated the disparity maps using these enhanced downsampled left camera images and the right camera images. 

The rectification process in the disparity map estimation changes the view of the rectified stereo images and each rectification by the three methods could have a slightly different geometric transformation. The view of the rectified images could thus vary for each of the three investigated methods and the groundtruth case. As a result of of registration issues in the pansharpening-based method, the variation in the view of the rectified images is even greater in the pansharpening-based method with respect to the other two methods. In order to have a fixed view that align the estimated disparity maps and also allow to conduct performance comparisons, the estimated disparity maps using the rectified images are warped to the left camera image view using the inverse rectification transformation matrix that was initially applied in the rectification process. The overlapping sections in the warped disparity maps (from the three methods and the groundtruth) to the left camera image view are considered as a mask and the pixels in this mask are used only to compute the average absolute error measure for the disparity map.

**Figure 17:**
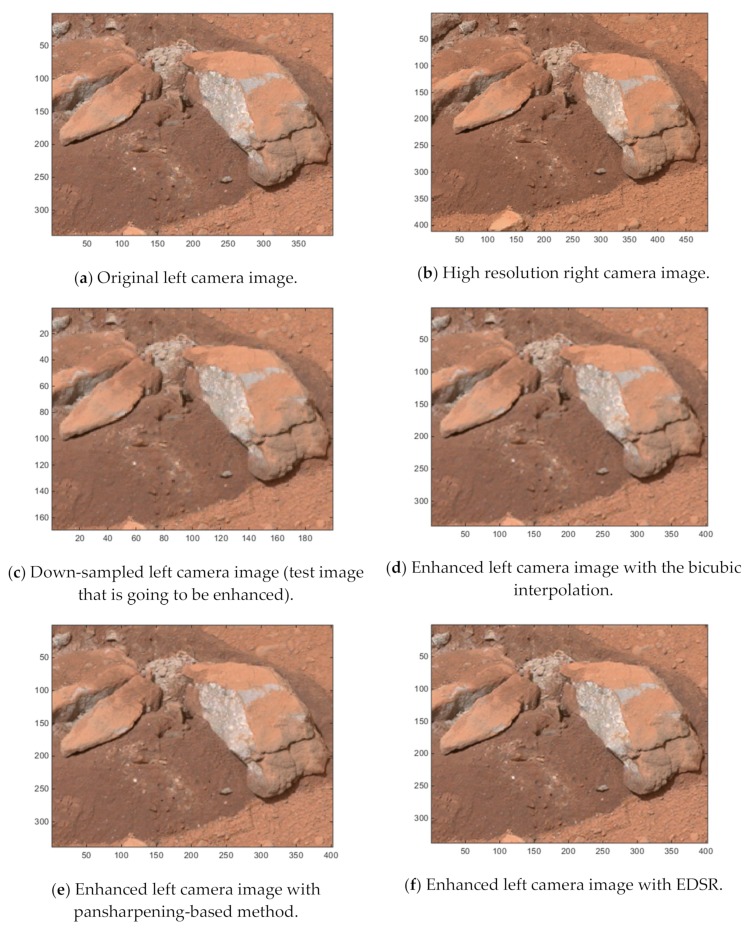
Mastcam image pair 6 (Sol 174) and the left camera image enhancements with the three methods.

Since the two-step image registration in the pansharpening-based method utilizes the random sample consensus (RANSAC) algorithm [27], each simulation run with the pansharpening-based method could generate slightly different results. To reduce the effects of this slight variation when assessing the applied measures, for each of the 20 Mastcam image pairs, we repeated the simulations 10 times and averaged the image quality scores and the average absolute errors (disparity map) with the pansharpening-based method. As a demonstration of the enhancements with the three methods, Figure 17a,b show the original left and right Mastcam images for one of the 20 investigated image pairs (Mastcam image pair 6). Figure 17c corresponds to the test image (down-sampled left camera image) which is going to be enhanced with the three methods and Figure 17d–f correspond to the enhanced left camera images with the three methods. 

**Figure 18:**
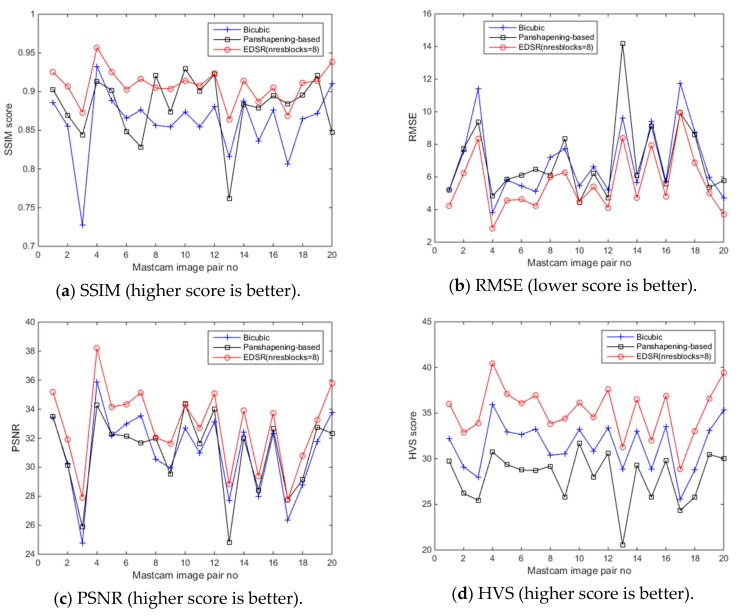

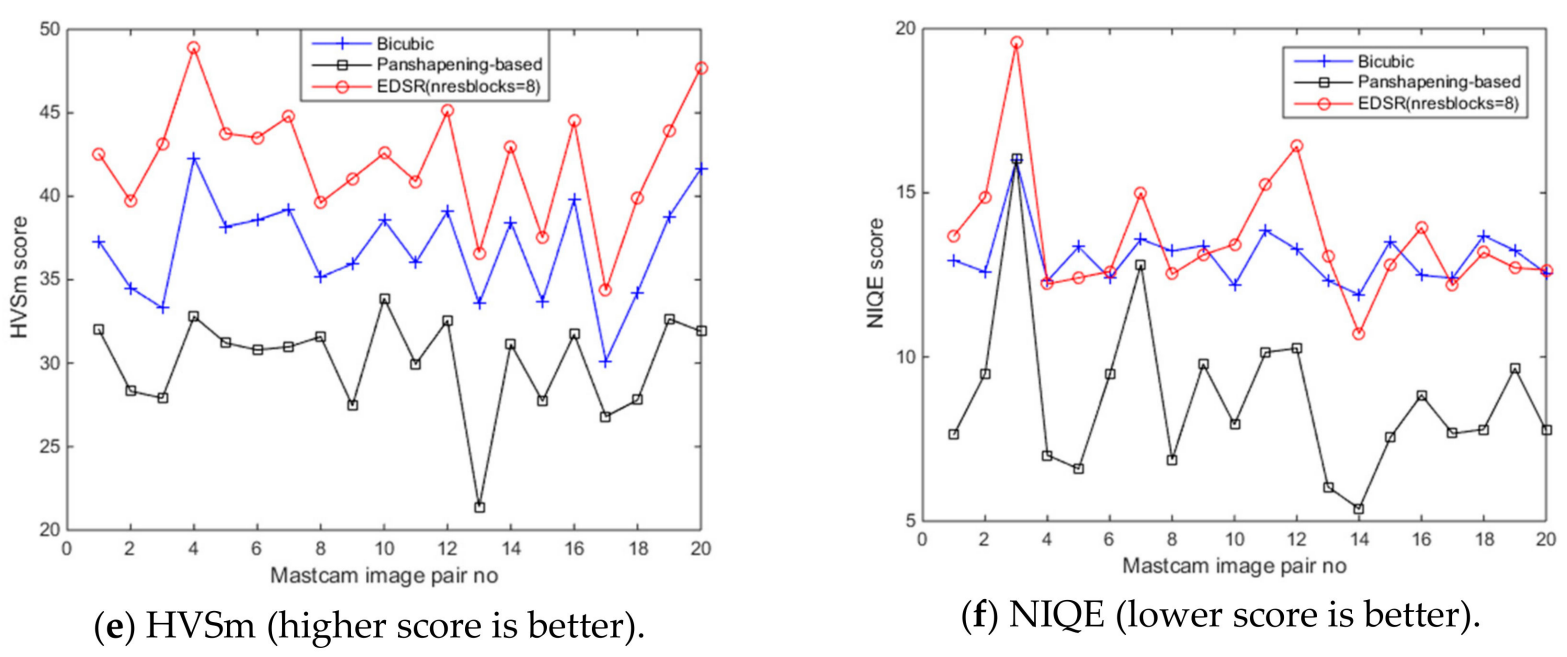
Enhancement comparisons with six image quality measures for the 20 Mastcam image pairs.

SSIM, RMSE, PSNR, HVS and HVSm values are computed for measuring the image quality of the enhanced “down-sampled left Mastcam images” with respect to the ground truth left camera image. Additionally, the NIQE measure is also applied. The plots of the six image quality values for 20 enhanced “downsampled Mastcam left camera images” can be seen in Figure 18. Overall, EDSR performs better than the other two methods in five of the six measures with the exception of NIQE. One interesting observation is that with SSIM, there are three image pairs (out of 20) where the pansharpening-based method performed better than EDSR. As was earlier noticed in the motorcycle image pair, the pansharpening-based method’s performance is affected due to some image pixel sections not aligned well with the two-step registration technique. We also notice this from the low scores in the HVS and HVSm measures. Other than the registration issues with the pansharpening-based method that affect some of these image quality measures negatively (except NIQE), it is also worth mentioning that because the original left camera images are used as the ground truth when computing the image quality measures and that the pansharpening-based method contains more detailed information in its enhanced left camera images which cannot be even visually noticed in the ground truth image (original left camera image), some of the applied image quality scores (except NIQE) for the pansharpening-based method look generally poor. Yet, in several of the image pairs, the RMSE and PSNR scores of the pansharpening-based method are still slightly better than the bicubic interpolation. With respect to the NIQE measure, the pansharpening-based method outperforms the other two significantly. This was predictable since the enhanced left camera images with the pansharpening-based method are more appealing to the eye with sharper details when compared to the EDSR and bicubic interpolation. This can be seen from the example images in Figure 12, Figure 13 and Figure 14, and also in Figure 16.

**Figure 19:**
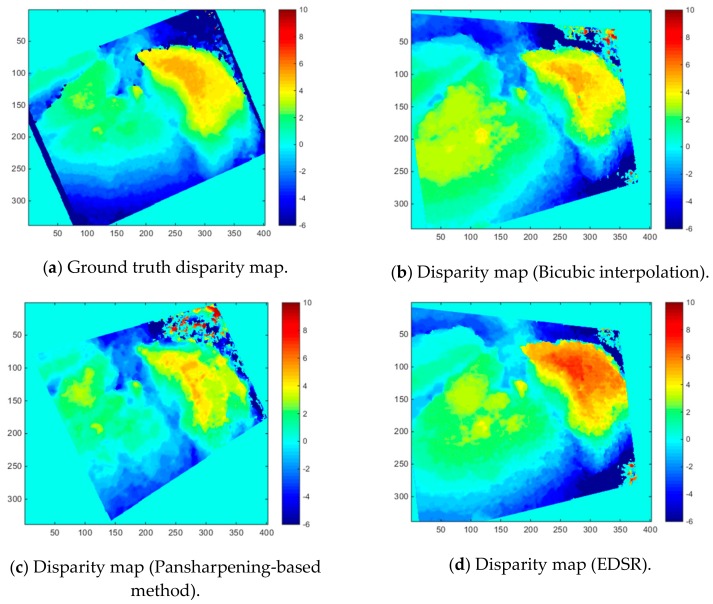

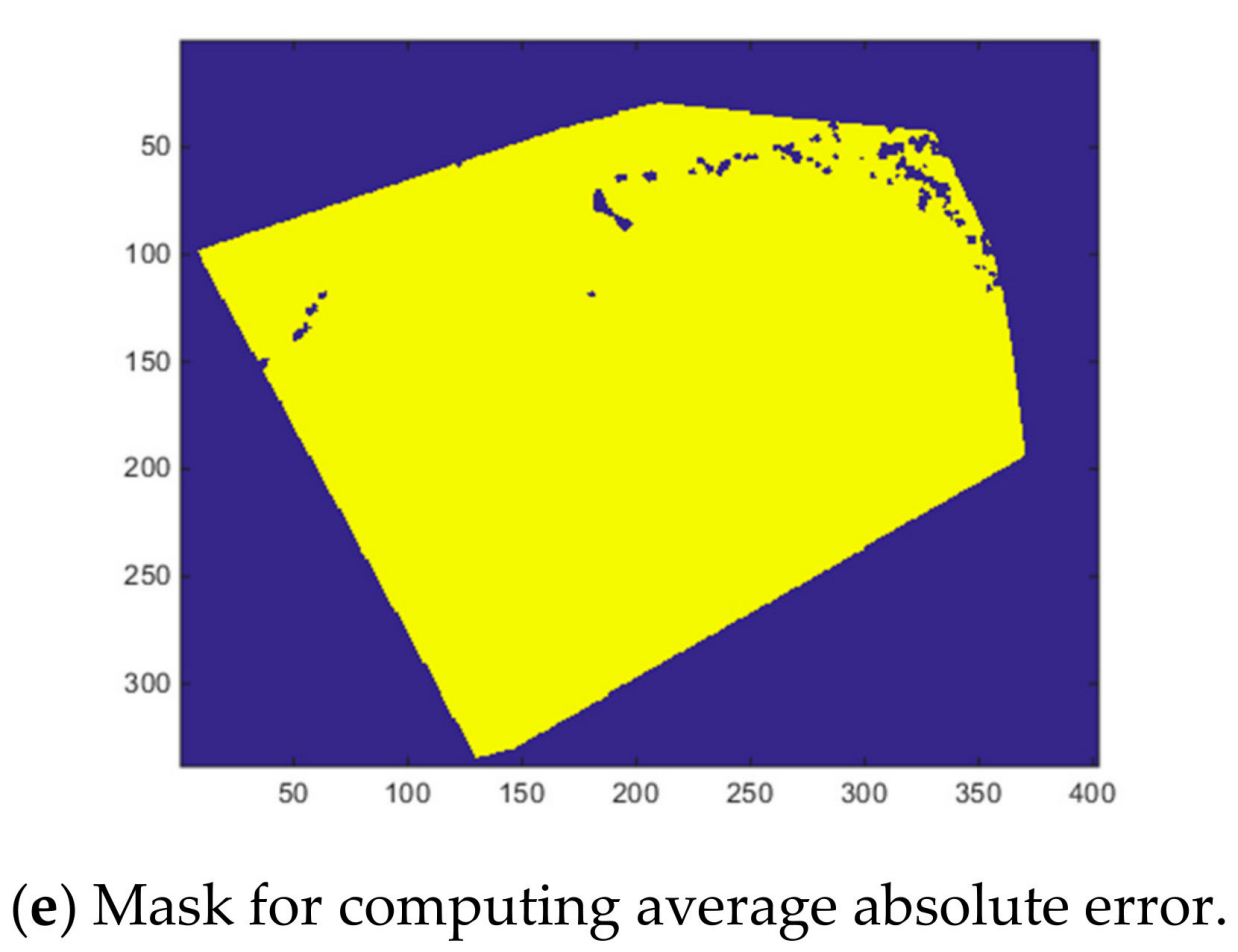
Disparity map estimations with the three methods and the mask for computing average absolute error.

Figure 19 demonstrates the disparity map estimations with the three methods for one of the 20 Mastcam image pairs (Image pair 6). Figure 19a corresponds to the estimated disparity map obtained with using the original left camera image which is considered as the ground truth disparity map in the first iteration. Figure 19b–d show the resultant disparity maps with the three methods. Figure 19e corresponds to the mask used when computing the average absolute error values. In order to give an idea about the number of matched SURF features in Figure 19, Table 8 shows the number of matched SURF features used in the disparity map estimation for this image pair. 

**Figure 20:**
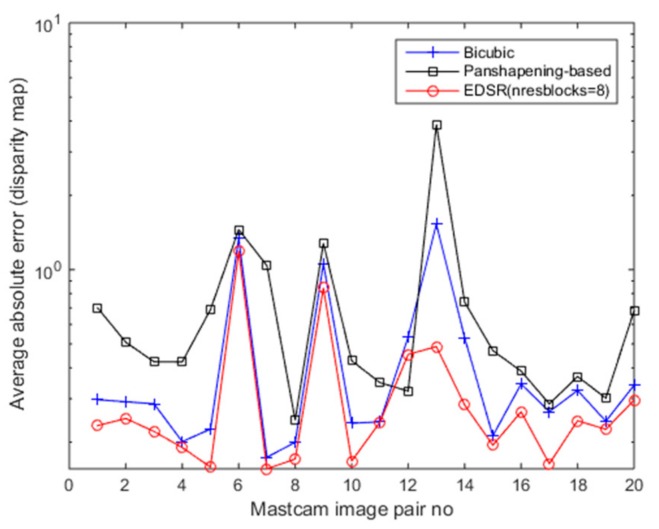
Average absolute errors for 20 Mastcam image pairs.

Figure 20 shows the average absolute error value plots for the disparity map estimates of the 20 Mastcam image pairs with the three methods. Among the three methods, the average absolute error value is lowest for the EDSR and the average absolute error values were found considerably higher for the pansharpening-based method. In only one image pair (out of 20) the pansharpening-based method provided the lowest average absolute error value. It can be seen that overall EDSR improves the left camera image quality better than the other two methods and the disparity map estimations using the EDSR enhanced left camera images are also better according to the average absolute error measure (in 19 of 20 Mastcam image pair EDSR performed better than the other two methods). EDSR works better than the other two methods since it does not have registration issues that the pansharpening method has and also does not have the blurring issues that the bicubic interpolation method has. From the viewpoint of its architecture, it is also based on ResNet with residual blocks in its architecture. Deep learning architectures with residual blocks when compared to other architectures are found to show better generalization and better efficiency [40]. All these attributes of EDSR positively affect the SURF feature extraction process in the disparity map estimation and enables finding SURF features in the enhanced left camera image that match better to the SURF features extracted in the right camera images. However, it is our thinking that if the pansharpening-based method had sub-pixel level registration for the whole image, it was highly likely that it would have performed better than EDSR since the enhanced left camera images by the pansharpening-based method look visually superior to the EDSR enhanced images. 

## 7. Conclusions

This paper introduced an in-depth study for the high-resolution disparity map estimation using the stereo Mastcam images with different resolutions acquired from the right and left Mastcam imagers of the Mars Curiosity rover. Among the three investigated methods, it is observed that the deep learning-based method, EDSR, had a better image resolution enhancement performance than the pansharpening-based method and bicubic interpolation. The impact of the low-resolution image enhancement on the disparity map estimation is examined and it is found out that a high resolution thus a more accurate disparity map estimation could be obtained after enhancing the low-resolution left camera image with EDSR. The pansharpening-based method, which is an adaptation of the two-step image registration technique, is observed to provide visually very appealing images as this was also confirmed quantitatively from the resultant NIQE measures. However, the performance of the pansharpening-based method heavily depends on the registration accuracy of the stereo images, which can be difficult when the image scene in the stereo image pair has varying depth of field. Other than improving the quality of the stereo products and 3D scene reconstruction in the Mastcam imagery, this work can also benefit to cellphones with dual cameras. Enhancing the low-resolution camera image in a dual camera cellphone setup can certainly result in better quality 3D cell phone imagery while reducing the cost of the cellphone. That is, instead of using two sophisticated cellphone cameras, the cellphone manufacturers can use only one high-resolution camera while enhancing the cheaper low-resolution cellphone camera via image enhancement.

## Figures and Tables

**Table 1:** Components of the single-scale baseline EDSR architecture.

***Head:** Sequential(* *(0): Conv2d(3, 64, kernel_size=(3, 3), stride=(1, 1), padding=(1, 1))* ) ***Body:** 16 x ResBlock* *ResBlock(* *(body): Sequential(* *(0): Conv2d(64, 64, kernel_size=(3, 3), stride=(1, 1), padding=(1, 1))* *(1): ReLU(inplace)* *(2): Conv2d(64, 64, kernel_size=(3, 3), stride=(1, 1), padding=(1, 1))* * )* *)* ***Tail:** Sequential(* *(0): Upsampler ( (0): Conv2d(64, 256, kernel_size=(3, 3), stride=(1, 1), padding=(1, 1))* *(1): PixelShuffle(upscale_factor=2))* *(1): Conv2d(64, 3, kernel_size=(3, 3), stride=(1, 1), padding=(1, 1))* *)*

**Table 2:** Performance metrics for the enhanced left camera images with the ground truth left camera image as the reference image (the grayscale versions of the images are used in computing SSIM and RMSE). Bold format indicates the method providing better results.

Method	SSIM	RMSE	PSNR	HVS	HVSm
Bicubic interpolation	0.8844	9.3966	28.6328	23.2014	24.6190
Pansharpening-based method	0.9215	7.7628	30.2594	25.9768	27.0790
EDSR (Deep learning-based)	**0.9443**	**5.8523**	**32.6983**	**28.2295**	**30.5052**

**Table 3:** Average absolute error on disparity maps for motorcycle image section (pixels that have negative values in the ground truth disparity map are excluded from the analysis, pixels with negative values in the estimated disparity maps are set to zero). Bold format indicates the method providing better results.

	Average Absolute Error
Bicubic interpolation	8.7456
Pansharpening-based method	17.5770
EDSR (Deep learning-based method)	**5.1492**

**Table 4:** Image quality measures for the foreground image part of the enhanced left camera images. Bold format indicates the method providing better results.

Method	SSIM	RMSE	PSNR	HVS	HVSm
Bicubic interpolation	0.8600	10.6346	27.5653	22.1325	23.5467
Pansharpening-based method	0.9332	7.5230	30.5221	26.2792	27.7185
EDSR (Deep learning-based)	**0.9343**	**6.6221**	**31.6408**	**27.1654**	**29.4234**

**Table 5:** Average absolute error on disparity maps for the foreground image part of the motorcycle image section. Bold format indicates the method providing better results.

Method	Average Absolute Error
Bicubic interpolation	6.0159
Pansharpening-based method	3.2810
EDSR (Deep learning-based method)	**3.2040**

**Table 6:** List of 20 Mastcam image pairs used.

Mastcam Image Pair no	Left Camera Image Filename	Right Camera Image Filename
1	0013ML0000120000100169E01_DRCX_0PCT.png	0013MR0000120000100039E01_DRCX_0PCT.png
2	0013ML0000120070100176E01_DRCX_0PCT.png	0013MR0000120070100046E01_DRCX_0PCT.png
3	0023ML0001140700100703C00_DRCX_0PCT.png	0023MR0001140700100600C00_DRCX_0PCT.png
4	0150ML0008420000104432E01_DRCX_0PCT.png	0150MR0008420000201218E01_DRCX_0PCT.png
5	0172ML0009240020104881E01_DRCX_0PCT.png	0172MR0009240020201683E01_DRCX_0PCT.png
6	0174ML0009350000105177E01_DRCX_0PCT.png	0174MR0009350070201948E01_DRCX_0PCT.png
7	0183ML0009930000105284E01_DRCX_0PCT.png	0183MR0009930070202041E01_DRCX_0PCT.png
8	0184ML0009250350105335E01_DRCX_0PCT.png	0184MR0009250350202097E01_DRCX_0PCT.png
9	0192ML0010170000105681E01_DRCX_0PCT.png	0192MR0010170000202484E01_DRCX_0PCT.png
10	0269ML0011810000106129E01_DRCX_0PCT.png	0269MR0011810000203215E01_DRCX_0PCT.png
11	0275ML0011960010106210E01_DRCX_0PCT.png	0275MR0011960010203447E01_DRCX_0PCT.png
12	0290ML0012250030106375E01_DRCX_0PCT.png	0290MR0012250030203531E01_DRCX_0PCT.png
13	0300ML0012410000106432E01_DRCX_0PCT.png	0300MR0012410000203739E01_DRCX_0PCT.png
14	0301ML0012530020106447E01_DRCX_0PCT.png	0301MR0012530020203760E01_DRCX_0PCT.png
15	0303ML0012610000106474E01_DRCX_0PCT.png	0303MR0012610000203818E01_DRCX_0PCT.png
16	0308ML0012730400106645E01_DRCX_0PCT.png	0308MR0012730400204006E01_DRCX_0PCT.png
17	0508ML0020000260202787E01_DRCX_0PCT.png	0508MR0020000260303151E01_DRCX_0PCT.png
18	0514ML0020280000202963E01_DRCX_0PCT.png	0514MR0020280000303241E01_DRCX_0PCT.png
19	0803ML0035130050400877E01_DRCX_0PCT.png	0803MR0035130050500252E01_DRCX_0PCT.png
20	0813ML0035700050401024E01_DRCX_0PCT.png	0813MR0035700050500419E01_DRCX_0PCT.png

**Table 7:** Average of image quality measures for 20 enhanced “down-sampled (×2) left” camera images with four EDSR models. Bold format indicates the method providing better results.

	EDSR Model (n_resblocks = 4)	EDSR Model (n_resblocks = 8)	EDSR Model (n_resblocks = 16)	EDSR Model (n_resblocks = 32)
SSIM	0.90674	**0.908093**	0.902667	0.901051
RMSE	5.670515	**5.632352**	5.842843	5.898821
PSNR	32.75032	**32.79714**	32.51517	32.42496
HVS	35.16401	**35.21195**	34.80805	34.75265
HVSm	42.06746	**42.14309**	41.29008	41.22219

**Table 8:** Number of matching SURF features used in the disparity map estimation.

Mastcam Image Pair no	Groundtruth	Bicubic Interpolation	Pansharpening-Based	EDSR (nresblocks = 8)
1	49	46	44	36
2	201	154	177	134
3	79	58	69	44
4	117	107	114	102
5	200	162	197	159
6	90	70	86	73
7	80	71	82	33
8	223	177	204	176
9	155	119	159	117
10	89	71	81	61
11	97	81	88	68
12	76	75	77	54
13	229	165	188	172
14	176	142	173	139
15	238	179	215	165
16	134	115	126	100
17	344	269	327	277
18	188	147	176	140
19	114	104	114	109
20	116	108	114	98
